# Beneficial effects of L-ornithine L-aspartate for prevention of overt hepatic encephalopathy in patients with cirrhosis: a systematic review with meta-analysis

**DOI:** 10.1007/s11011-019-00463-8

**Published:** 2019-07-23

**Authors:** Roger F. Butterworth

**Affiliations:** Department of Medicine, University of Montreal, 45143 Cabot Trail, Englishtown, Nova Scotia, B0C 1H0 Canada

**Keywords:** L-ornithine L-aspartate, Hepatic encephalopathy, Overt hepatic encephalopathy, Cirrhosis, Systematic review, Meta-analysis, Prevention, Primary prophylaxis, Secondary prophylaxis, Post-TIPSS prophylaxis

## Abstract

The present systematic review with meta-analysis was undertaken to review the evidence base in support of a beneficial effect of L-ornithine L-aspartate (LOLA) for the prevention/prophylaxis of overt hepatic encephalopathy (OHE) in patients with cirrhosis. Using appropriate keywords and electronic and manual searches together with established inclusion/exclusion criteria, six randomized controlled trials (RCTs) for a total of 384 patients were identified five of which were of high quality and low risk of bias according to Jadad-Cochrane criteria. Treatment with LOLA resulted in significant reductions in the risk of progression to OHE in MHE patients (3 studies) with RR: 0.23 [95% CI: 0.07, 0.73], *p* < 0.01. LOLA was also effective for secondary OHE prophylaxis with RR: 0.389 [95% CI: 0.174–0.870] *p* < 0.002 as well as for primary prophylaxis for OHE following acute variceal bleeding [RR: 0.42 [95% CI: 0.16–0.98] *p* < 0.03 and for OHE prophylaxis post-TIPSS [RR: 0.30 [95% CI: 0.03–2.66] compared to placebo/no intervention in all cases. OHE prevention/prophylaxis was accompanied by significant reductions of blood ammonia. Both oral and intravenous formulations of LOLA appeared to be effective for the prevention of progression to OHE in patients with MHE. These findings provide the first direct evidence of potential benefit of LOLA for the prevention of OHE in cirrhosis across a range of clinical presentations.

## Introduction

Hepatic encephalopathy (HE) is a serious central nervous system (CNS) complication of cirrhosis characterized by a spectrum of neurological and neuropsychiatric symptoms whose appearance heralds a deteriorating medical condition. Clinically manifest or overt hepatic encephalopathy (OHE) starts with disorientation and lethargy that gives way to asterixis followed ultimately by stupor and coma. The presence of OHE has a negative impact on quality of life as well as on patient survival. In addition, the presence of OHE has a significant negative impact on neurocognitive function before (Wong et al. 2014) and after (Sotil et al. 2009) liver transplantation. Moreover, each bout of OHE is associated with increased risk of further OHE episodes (Weissenborn 2019). In light of the above considerations, effective approaches aimed at the prevention of OHE in cirrhosis are urgently required.

Hyperammonemia is a consistently-reported feature of OHE in patients with cirrhosis and the current mainstay of therapy involves the use of agents whose actions are aimed at the lowering of circulating ammonia (Butterworth 2019). This is accomplished by one of two principal approaches aimed either at the reduction of ammonia production in the gut (non-absorbable disaccharides or antibiotics) or increased ammonia removal by residual hepatocytes or skeletal muscle cells using L-ornithine L-aspartate (LOLA). Beneficial effects of LOLA have been reported in reviews of over 20 randomized controlled trials (Butterworth et al. 2018) and meta-analyses (Butterworth and McPhail 2019) for the treatment of not only OHE but also for the early pre-symptomatic (minimal or covert) forms of the disorder where oral formulations of LOLA appear to be particularly effective.

However, the use of LOLA for the prevention/prophylaxis of OHE in cirrhosis has not, to date, been a major source of concern and there are no published reports of systematic reviews/meta-analyses of trials dedicated to the efficacy of LOLA for OHE prevention. Sporadic reports are limited in number to small sub-groups of very low numbers of trials and patients but results so far are inconsistent (Goh et al. 2018; Thumburu et al. 2017). Given this paucity of available data and as a basis for planned research in this area, the present systematic review with meta-analysis was undertaken in order to review the evidence base in support of a beneficial effect of LOLA for OHE prevention/prophylaxis in patients with cirrhosis. Criteria for searches and inclusion/exclusion as well as assessment of quality, bias and heterogeneity of trials were as previously described in detail (Butterworth et al. 2018), a summary of which is provided in Methods below.

## Methods

### Search criteria

Manual and electronic searches were made with appropriate keywords (hepatic encephalopathy, cirrhosis, L-ornithine L-aspartate, LOLA, prevention, prophylaxis, controlled trial) of listings in Medline, PubMed, Cochrane controlled trials register (2008), Google search and Clinical trials.gov in English, French, German or other languages with available translations.

### Inclusion/exclusion criteria

Trials involving males or females over 18 years of age were included. The analysis aimed to compare effects of LOLA (oral or intravenous formulations) compared to placebo/no intervention in randomized controlled clinical trials (RCTs) with adequate description of patient characteristics, blinding of personnel, patients, investigators, numbers of dropouts, dose and route of administration of LOLA or placebo in sufficient detail for assessment of trial quality and risk of biases as previously reported (Butterworth et al. 2018).

Trials that were uncontrolled, open-label, observational, involving non-cirrhotic patients, acute liver failure, publication of results in abridged form (review, abstract, editorial, conference proceedings) were excluded unless adequate details for assessment of outcome, risk of bias were provided.

### Outcome measures

The primary outcome measure was defined as the prevention of an episode of OHE in patients with cirrhosis as the first episode (primary prophylaxis), a repeat episode (secondary prophylaxis), prevention of progression of MHE to OHE or prevention of an episode of OHE occurring post-transjugular intrahepatic stent shunt (TIPSS), a procedure employed to treat complications of cirrhosis (portal hypertension, refractory ascites).

### Assessments of trial quality, bias and heterogeneity

Use was made of a custom-designed assessment paradigm in which elements of the Jadad composite scale (Jadad et al. 1996) together with that of the reporting items for systematic reviews and meta-analysis protocols (PRISMA-P) 2015 statement (Moher et al. 2009). Assessments of the risk of bias made use of the Cochrane Collaboration’s tool for assessment of bias relating to selection, performance, detection, attention and reporting of the main outcome (Higgins and Green 2011). Bias related to publication of trial results was assessed by regression analysis. In the present analysis, overall trial quality was considered to be high for trials with Jadad scores of 3 and above and/or low risk of bias according to the Cochrane tool.

### Statistical analyses

For continuous variables, groups were compared by mean differences with 95% confidence intervals. For dichotomous variables, the relative risk (RR) was considered with 95% confidence intervals. Heterogeneity was assessed using the x^2^ test with significance set at *p* < 0.10. Since RR results in similarly consistent results to that of the Odds Ratio (OR), RR was used for dichotomous variables in order to facilitate comparisons with results from previously-reported analyses. Aggregations of the results of the primary studies were made using the Random Effects model in all cases.

## Results

### Trial selection

Electronic searches of databases identified 43 trials with a further 16 trials from manual searches. Five full-text articles were excluded due to incompatibility of data required for pooling. Removal of duplicate citations and elimination of studies in line with inclusion/exclusion criteria resulted in 6 trials for inclusion in the final analysis where sufficient data were available.

Four cirrhosis-related clinical presentations of OHE were identified in which details of LOLA-related prevention/prophylaxis were provided namely the prevention of the progression of minimal HE (MHE) to OHE, the prevention of recurrence of OHE (secondary prophylaxis), primary prophylaxis aimed at the prevention of OHE associated with acute variceal bleeding and the prevention of OHE associated with the TIPSS procedure. A summary of the characteristics of included trials is provided in Table 1.

**Table 1:** Characteristics of included trials

Trial reference	Patient numbers	LOLA dose	Route of administration
Mittal et al. 2011	LOLA *n* = 40, Pla n = 40	18 g/d, 3Mo	po
Abid et al. 2011	LOLA *n* = 6, Pla *n* = 6	20 g/d, 3d	iv
Alvares-da-Silva et al. 2014	LOLA *n* = 28, Pla *n* = 35	15 g/d, 60d	po
Varakanahalli et al. 2018	LOLA *n* = 73, Pla *n* = 72	6 g/tid, 6Mo	po
Higuera-de-la-Tijera et al. 2018	LOLA n = 22, Pla *n* = 22	10 g/d, 7d	iv
Bai et al. 2014	LOLA *n* = 21, Pla *n* = 19	30 g/d, 7d	iv

Trials are identified by first author and year [with reference number in parentheses]. *Pla* placebo, *po* oral formulation, *iv* intravenous formulation

### Quality and risk of bias for included trials

Trial quality and risk of bias assessments performed according to the combined Jadad/Cochrane procedure described in Methods (above) are shown in Table 2. Trial quality relating to the assessment of randomization, blinding and information related to drop-outs was judged to be of high quality (Jadad scores of 3 or more) (Table 2) in all included trials.

**Table 2:** Quality and Bias Assessment of included trials

	Mittal et al. 2011	Abid et al. 2011	Alvares-da-Silva et al. 2014	Varakanahalli et al. 2018	Higuera-de la Tijera et al. 2018	Bai et al. 2014
A. Jadad Score						
Randomization	2	1	2	2	2	2
Blinding	0	1	1	2	2	2
Dropouts	1	1	1	1	1	1
Total score	**3**	**3**	**4**	**5**	**5**	**5**
B. Cochrane Score						
Selection bias (randomization)	L	L	L	L	L	L
Selection bias (blinding)	L	L	L	L	L	L
Performance Bias	H	L	L	L	L	L
Detection bias	H	UC	L	L	L	L
Reporting bias	L	L	L	L	L	L
Overall bias	**UC**	**L**	**L**	**L**	**L**	**L**

Trial quality is indicated by a score of 1–5 on the Jadad scale; scores of 3 or above indicate high quality. Assessment of risk of bias due to randomization, blinding, detection and attrition using the Cochrane tool for risk of bias assessment as described in Methods. *L* low, *H* high, *UC* unclear

Risk of bias assessment was judged to be low in 4/6 trials and unclear in the remaining 2 trials due principally to detection bias (2 trials) and performance bias (1 trial) evaluated using the Cochrane tool for bias assessment (Table 2); there was no evidence of publication bias in any of the selected trials.

### Meta-analysis: efficacy of LOLA versus placebo/no intervention for prevention of OHE

**Fig. 1:**
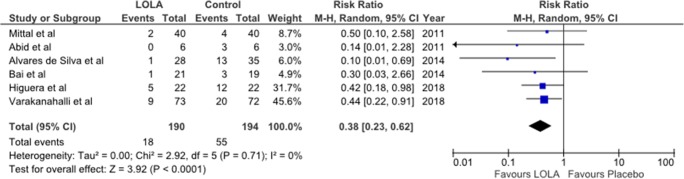
Forest Plot for the efficacy of LOLA versus placebo/no intervention for the prevention of progression of MHE to OHE (Abid et al. 2011; Mittal et al. 2011; Alvares-da-Silva et al. 2014), secondary OHE prophylaxis (Varakanahalli et al. 2018), primary OHE prophylaxis (Higuera-de-la-Tijera et al. 2018) or post-TIPSS OHE prophylaxis (Bai et al. 2014) from results of the appropriate published RCTs. Trials are identified by first author names, number of the reference in parentheses and year of publication

Forest plots indicating the pooled effect in 384 patients of the efficacy of LOLA compared to placebo/no intervention for the prevention of OHE regardless of the clinical OHE subtype are provided in Fig. 1. Assessment of the pooled data revealed that LOLA was consistently more effective compared to placebo/no intervention in all 6 trials [with RR of 0.38, 95% CI of 0.23–0.62], test for overall effect, Z = 3.92, *p* < 0.0001]. Both intravenous and oral formulations of LOLA appeared to be effective but trial and patient numbers were insufficient in number for the quantitative independent assessment of efficacy of the two formulations.

#### Prevention of progression of MHE to OHE (subgroup analysis)

**Fig. 2:**
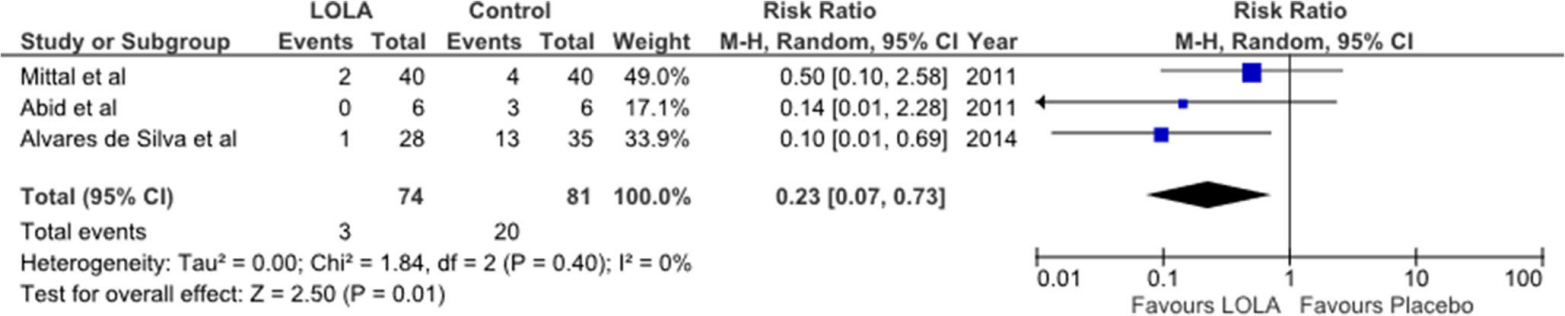
Forest plot for the efficacy of LOLA versus placebo/no intervention for the prevention of progression from MHE to OHE in patients with cirrhosis from results of published RCTs. Trial identification as for legend to Fig. 1

Three RCTs were identified in which the efficacy of LOLA was studied compared to placebo/no intervention for the prevention of deterioration of MHE to OHE. Results of Forest Plots are summarized in Fig. 2.

The first such trial (Mittal et al. 2011) compared lactulose, probiotics and LOLA for the treatment of MHE in which 80 patients with cirrhosis and MHE diagnosed by psychometric testing were randomized to receive no treatment (*n* = 40) or LOLA orally 18 g/d for 3 months (n = 40) the end point being the progression to OHE. 4/40 patients developed OHE in the no treatment group compared to 2/40 in the LOLA treatment group [RR: 0.50 95% CI: 0.10–2.58] and this was accompanied by significant improvements in HRQOL. Findings of comparable efficacy were observed following treatment with probiotics or lactulose.

In the second trial, (Abid et al. 2011) 6 patients with MHE were given intravenous LOLA (20 g/4 h for 3 consecutive days) or placebo (*n* = 6). Deterioration of MHE assessed by West Haven criteria showed that 3/6 (50%) in the placebo group manifested deterioration to OHE compared to 0/6 (0%) in the LOLA treatment group [RR: 0.14 95% CI: 0.01–2.28].

In the third trial (Alvares-da-Silva et al. 2014) of the efficacy of the oral formulation of LOLA for the prevention of OHE, 64 patients with cirrhosis and MHE were treated with LOLA (5 g tid, 60d) or placebo. Patients in the LOLA group had significantly less episodes of OHE at 6 months (5% of 28 LOLA-treated patients compared to 37.9% of 35 patients receiving placebo [RR: 0.10, 95% CI: 0.01–0.69] *p* < 0.016).

In all three MHE subgroup trials described above, prevention of OHE was accompanied by significant decreases of blood ammonia consistent with the established mechanism of ammonia-lowering action of LOLA. Moreover, in the third trial, patients showing benefit from treatment with LOLA had evidence of improved liver function reflected in improvements of Child Pugh and MELD scores.

With regard to the prevention of progression of MHE to OHE in patients with cirrhosis, a recent systematic review and network meta-analysis of the comparative effectiveness of LOLA, lactulose, rifaximin, synbiotics, and branched-chain amino acids (BCAAs) were compared to placebo/no intervention for the prevention of development of OHE (Thumburu et al. 2017). LOLA and lactulose were found to be effective but rifaximin was not so. Data for the efficacy of LOLA: OR: 0.16, 95%CI (0.04–0.64).

#### Secondary OHE prophylaxis: prevention of recurrence of OHE

In a double-blind RCT, the effectiveness of LOLA (oral formulation) was studied on the recurrence of OHE in 150 patients with cirrhosis (Varakanahalli et al. 2018). Patients were randomized to receive LOLA (3 × 6 g/d) or placebo for 6 months. Secondary prophylaxis was defined as prevention of recurrence of an episode of OHE in patients who had manifested one or more previous episodes of OHE prior to treatment. Primary endpoints were the recurrence of OHE or a follow-up period of 6 months. The primary objective was the assessment of the superiority of LOLA over placebo in preventing OHE recurrence. Secondary objectives were time to first breakthrough episode of OHE, time to first OHE-related hospital admission, mortality, safety of continuous treatment, adverse events, changes in arterial ammonia, HE grading, improvements in HRQOL and predictors of OHE recurrence. On an intention-to-treat basis, 20 of the 72 patients in the placebo group developed OHE compared to 9 of 73 patients treated with LOLA giving an RR of 0.44 [95% CI: 0.22–0.91] *p* < 0.022.

**Fig. 3:**
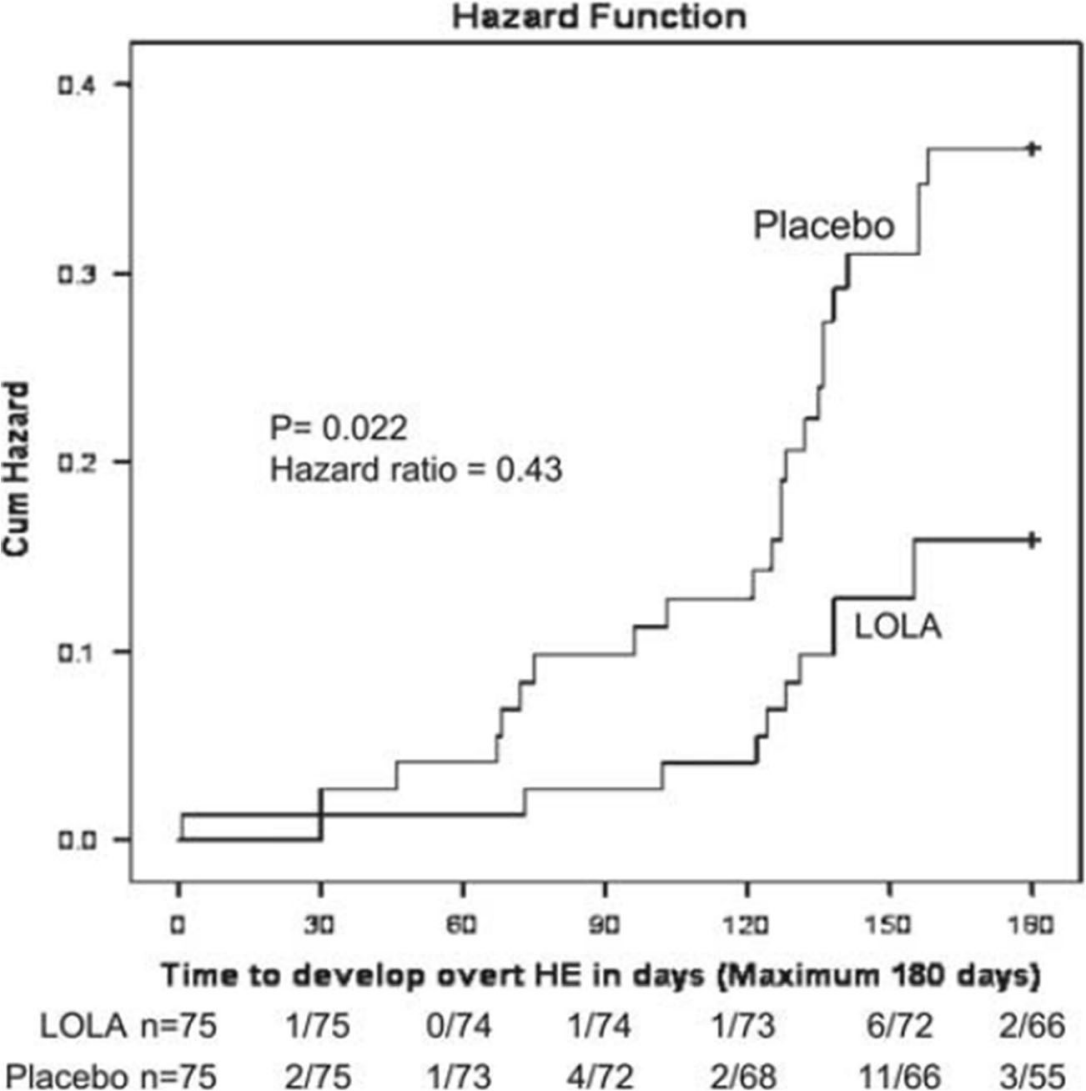
Secondary prophylaxis of OHE in patients with cirrhosis. Time for breakthrough of OHE is indicated from randomization to six month follow-up in LOLA treatment group compared to placebo by the Cox proportional hazard model resulting in HR of 0.431 [95% CI: 0.210–0.885], *p* < 0.022, from Varakanahalli et al. 2018 with permission

Time to first OHE breakthrough episode was 157.78 days [95% CI: 148.5–167.0] for placebo versus 170.88 days [95% CI: 165.0–176.73] in the LOLA treatment group with HR: 0.431 [95% CI: 0.210–0.885] p < 0.022 (Fig. 3). The probability of developing OHE in patients receiving LOLA was reduced by 37% compared to placebo and the number needed to prevent the first breakthrough episode of OHE was 6.25 on an intention-to-treat basis.

At 6-months follow-up, significantly greater reductions of arterial ammonia were observed in the LOLA treatment group (−23.58 ± 14.8 μmol/L) versus placebo (1.41 ± 13.34 μmol/L), *p* < 0.001.The time to first OHE-related hospital admission was longer in the LOLA group compared to placebo with *p* < 0.021 with concomitantly greater improvements in HRQOL (SIP scores) (p < 0.001). Predictors of recurrence of OHE on univariate analysis at 3-month follow-up in this study included baseline scores for Child-Turcotte-Pugh, MELD, CFF scores, PHES scores and arterial ammonia concentrations.

#### Primary OHE prophylaxis following acute variceal bleeding in patients with cirrhosis

Acute variceal bleeding is a major precipitating factor for episodic OHE in cirrhosis. However current guidelines for the prevention of OHE under these conditions are scant. In order to address this issue, a placebo-controlled RCT was undertaken in 87 patients with cirrhosis and acute variceal bleeding in order to compare the efficacy of primary prophylaxis using one of three agents namely lactulose, LOLA and rifaximin, compared to placebo (Higuera-de-la-Tijera et al. 2018). The primary endpoint was the development of OHE in the 7-day period post-bleeding. Secondary end points were the time in days for the first appearance of OHE as well as the late occurrence of OHE in the ensuing 28 day period. The LOLA treatment group received intravenous infusions of LOLA (10 g/24 h for 7 days).

The frequency of development of OHE with intravenous LOLA treatment was 22.7% compared to 54.5% with placebo [OR: 0.2, 95% CI: 0.06–0.88, *p* < 0.03]. The relative severity of OHE grade assessed by West Haven criteria was: for placebo: median Grade 3, Range 2–4, for LOLA: median Grade 1, Range 1–2, *p* < 0.04. Effective prophylaxis of a comparable magnitude to those following LOLA treatment was observed in the rifaximin treatment group whereas results using lactulose fell below the threshold for statistical significance. No adverse events or deaths were registered in patients in the LOLA treatment group.

#### OHE prophylaxis post-TIPSS

New or worsening episodes of OHE occur in up to 50% of patients with cirrhosis following the TIPSS procedure for the treatment of complications of portal hypertension (Rossle et al. 1994). Results of a study published in 2005 showed that neither rifaximin nor lactitol were effective for HE prophylaxis post-TIPSS (Riggio et al. 2005).

However, results of a subsequent RCT of 40 patients revealed that LOLA infusions of 30 g/day for 7 consecutive days was effective for prevention of progression from MHE to OHE in patients with TIPSS: [RR of 0.30 95% CI: 0.03–2.66] and improvement following LOLA treatment occurred in parallel with decreases in fasting and post-prandial venous ammonia concentrations (Bai et al. 2014). Moreover, these benefits were accompanied by improvements in circulating levels of liver enzymes and bilirubin as well as MELD scores consistent with the notion that the 7-day LOLA treatment paradigm resulted in the reduction of post-TIPSS hepatic injury. These findings confirm and extend previous reports of hepato-protective properties of LOLA in patients with cirrhosis (Mittal et al. 2011; Alvares-da-Silva et al. 2014; Chen et al. 2005). Novel hepato-protective mechanisms involving the production of anti-oxidants and improvements in hepatic microcirculation have been proposed to explain these beneficial effects of LOLA in patients with cirrhosis (Butterworth and Gruengreiff 2019) and in patients with non-alcoholic fatty liver disease (Butterworth and Canbay 2019) via a similar mechanism of action.

#### Dose regimens for prevention of OHE by LOLA

Analysis of doses and duration of LOLA treatment were within previously-published ranges used extensively for OHE treatment. For example, prevention of progression of MHE to OHE in cirrhosis as well as secondary OHE prophylaxis made use of 5-6 g tid for periods of three to six months for the oral formulation (Mittal et al. 2011; Alvares-da-Silva et al. 2014; Varakanahalli et al. 2018). For intravenous infusion of LOLA, 20 g LOLA for 3 days was employed (Abid et al. 2011). The single primary prophylaxis trail employed intravenous LOLA 10 g over a 24 h period for 7 days, (Higuera-de-la-Tijera et al. 2018).

## Discussion and conclusions

Results of the present systematic review and meta-analysis demonstrate, for the first time, that LOLA is effective for the prevention of OHE in patients with cirrhosis in a relatively wide spectrum of clinical presentations including decreased progression of MHE to OHE, prevention of the recurrence of OHE / secondary prophylaxis, primary OHE prophylaxis associated with acute variceal bleeding and post-TIPSS OHE prophylaxis. Whether analyzed individually or collectively, LOLA treatment resulted in significant prevention of OHE and reduction of blood ammonia in the 5 trials in which it was measured. The majority of included trials were of high quality and low risk of bias according to Jadad-Cochrane guidelines. Both oral and intravenous formulations of LOLA appeared to be effective for lowering of the risk of progression of MHE to OHE but additional studies are necessary in order to confirm these findings.

Results of a previous network meta-analysis (Thumburu et al. 2017) are encouraging in which deterioration of MHE to OHE was significantly reduced following treatment with LOLA compared to placebo/no intervention with RR: 0.23, [95% CI: 0.07–0.73, *p* < 0.01]. Treatment with lactulose was also found to be effective but differences following treatment with rifaximin failed to attain statistical significance.

The present systematic review with meta-analysis is the first of its kind relating to the effects of LOLA specifically on OHE prevention/prophylaxis in patients with cirrhosis. As anticipated, there are some shortcomings. Numbers of trials and patient enrollments are small so that sub-grouping was restricted to analysis of the three trials plus one network meta-analysis of the efficacy of LOLA for the slowing of the deterioration of MHE to OHE. It is hoped that the findings of the present review may stimulate further clinical research into the use of LOLA for primary and secondary OHE prophylaxis as well as trials of the use of LOLA for post-TIPSS OHE prophylaxis for which there remains an important unmet clinical need.
